# Effect of Aprepitant for the Prevention of Chemotherapy-Induced Nausea and Vomiting in Women: A Randomized Clinical Trial

**DOI:** 10.1001/jamanetworkopen.2021.5250

**Published:** 2021-04-09

**Authors:** De-Shen Wang, Ming-Tao Hu, Zhi-Qiang Wang, Chao Ren, Miao-Zhen Qiu, Hui-Yan Luo, Ying Jin, William Pat Fong, Shu-bin Wang, Jie-wen Peng, Qing-feng Zou, Qiong Tan, Feng-Hua Wang, Yu-Hong Li

**Affiliations:** 1 State Key Laboratory of Oncology in South China, Collaborative Innovation Center for Cancer Medicine, Sun Yat-sen University Cancer Center, Sun Yat-sen University, Guangzhou, People’s Republic of China; 2 Research Unit of Precision Diagnosis and Treatment for Gastrointestinal Cancer, Chinese Academy of Medical Sciences, Guangzhou, People’s Republic of China; 3 Department of Medical Oncology, Sun Yat-sen University Cancer Center, Guangzhou, People’s Republic of China; 4 Department of Oncology, Peking University Shenzhen Hospital, Shenzhen, People’s Republic of China; 5 Chemotherapy Department, Zhongshan People’s Hospital, Zhongshan, Guangdong Province, People’s Republic of China; 6 Section 3 of Internal Medicine, Cancer Center of Guangzhou Medical University, Guangzhou, Guangdong, People’s Republic of China

## Abstract

**Importance:**

The prevention of chemotherapy-induced nausea and vomiting has an important role in the overall management of cancer treatment.

**Objective:**

To evaluate whether adding aprepitant to palonosetron and dexamethasone can further prevent the incidence and severity of nausea and vomiting caused by FOLFIRI (fluorouracil, leucovorin, and irinotecan) or FOLFOX (fluorouracil, leucovorin, and oxaliplatin) chemotherapy regimens among women with gastrointestinal cancer at higher risk.

**Design, Setting, and Participants:**

This phase 3, double-blind, placebo-controlled randomized clinical trial recruited young women (age ≤50 years) who drank little or no alcohol and had gastrointestinal cancer for which they received FOLFOX or FOLFIRI chemotherapy. A total of 248 women were enrolled and assigned in the ratio 1:1 to intervention and control groups from August 4, 2015, to March 31, 2020. Intention-to-treat analysis was used to evaluate patient baseline characteristics and efficacy. The analysis was conducted on October 30, 2020.

**Interventions:**

Patients were randomly assigned to the aprepitant group (aprepitant, 125 mg, orally 60 minutes before initiation of chemotherapy on day 1 and 80 mg orally each morning of days 2 and 3; palonosetron, 0.25 mg, intravenously; and dexamethasone, 6 mg, orally 30 minutes before chemotherapy initiation on day 1) or the placebo group (placebo, 125 mg, orally 60 minutes before initiation of chemotherapy on day 1 and 80 mg orally on each morning of days 2 and 3; palonosetron, 0.25 mg, intravenously; and dexamethasone, 12 mg, orally 30 minutes before chemotherapy initiation on day 1).

**Main Outcomes and Measures:**

The primary end point was the complete response (CR) rate, defined as the proportion of patients without emesis episodes or rescue medication use during the overall phase of the first cycle. Other efficacy indicators, such as no vomiting and no nausea, were measured as the secondary and exploratory end points.

**Results:**

A total of 248 women from 4 clinical centers in China entered this study, and 243 patients (aprepitant regimen, 125 patients [51.4%]; placebo regimen, 118 patients [48.5%]) were evaluable for efficacy and safety; mean (SD) age of the total population was 40.1 (7.3) years. The CR rate was significantly higher in the aprepitant group vs the control group overall (107 [87.0%] vs 80 [66.7%]; *P* < .001) and in the acute (114 [92.7%] vs 91 [75.8%]; *P* = .001) and delayed (109 [88.6%] vs 84 [70.0%]; *P* = .001) phases of the trial. The incidence of adverse events was similar between the 2 groups (100 [80.0%] vs 96 [81.3%]; *P* = .79), and no grade 3 or 4 aprepitant treatment-related adverse events were observed. Multivariable analysis revealed that aprepitant use was the only independent factor associated with CR during the overall phase.

**Conclusions and Relevance:**

The combination of aprepitant with palonosetron and dexamethasone provided increased antiemetic efficacy in the FOLFOX or FOLFIRI chemotherapy regimen and was well tolerated by younger women with gastrointestinal cancer who have a history of little or no alcohol consumption.

**Trial Registration:**

ClinicalTrials.gov Identifier: NCT03674294

## Key Points

**Question:**

Is prophylactic aprepitant therapy combined with palonosetron and dexamethasone effective in preventing nausea and vomiting in women receiving moderately emetogenic chemotherapy?

**Findings:**

In this randomized phase 3 clinical trial that included 248 women younger than 50 years with no or little alcohol use, the complete response rate in the overall phase for chemotherapy-induced nausea and vomiting was 87% in the aprepitant group vs 67% in the control group. The overall complete response rate was significantly higher in the aprepitant group vs the control group.

**Meaning:**

Adding aprepitant to palonosetron and dexamethasone was effective in reducing nausea and vomiting for women who received moderately emetogenic chemotherapy.

## Teaser

This randomized clinical trial examines the use of aprepitant combined with palonosetron and dexamethasone for prophylactic treatment in women with chemotherapy-associated nausea and vomiting.

## Introduction

Chemotherapy-associated nausea and vomiting (CINV) are among the most common and distressing adverse effects in patients receiving antineoplastic therapy, which could seriously affect the quality of patients’ life and markedly reduce treatment adherence.^1,2,3^ Therefore, the prevention of CINV has a vital role in the overall management of anticancer treatment.

The incidence and severity of CINV depend mainly on the anticancer agents’ emetogenicity, which is divided into high, moderate, low, and mild emetogenic chemotherapy, according to the incidence of emesis in the absence of antiemetic prophylaxis.^4^ The FOLFOX (fluorouracil, leucovorin, and oxaliplatin) and FOLFIRI (fluorouracil, leucovorin, and irinotecan) regimens are used widely in treatment of gastrointestinal cancers. Oxaliplatin and irinotecan are classified as moderately emetogenic. Previous studies have shown that the vomiting incidence in patients who received FOLFOX or FOLFIRI as first-line therapy was more than 40% for both chemotherapeutic treatments.^4,5^ In addition to chemotherapy regimens, CINV has been associated with patient-related risk factors, such as female sex and young age. The incidence of vomiting in these patients has been shown to be high even though they received a standard 2-drug antiemetic regimen consisting of dexamethasone and palonosetron, a highly effective 5-hydroxytryptamine-3 (5-HT3) receptor antagonist (RA).^6^

Aprepitant is a neurokinin-1 (NK-1) RA, which has a different mechanism of action from that of 5-HT3 RA and other antiemetics used to prevent CINV.^7^ Aprepitant can selectively block the binding of substance P at the NK-1 receptor in the central nervous system, showing benefit in the control of both acute and delayed CINV. In patients receiving high-dose cisplatin-based or combination chemotherapy of anthracycline and cyclophosphamide, the triple-drug combination (NK-1 RA plus a standard regimen of 5-HT3 RA and dexamethasone) improved protection against CINV compared with the standard regimen.^8,9^ Thus, both the National Comprehensive Cancer Network guideline and the Multinational Association of Supportive Cancer Care (MASCC)/European Society for Medical Oncology (ESMO) guideline recommend prophylactic use of aprepitant in combination with a 5-HT3 RA and dexamethasone for patients receiving highly emetogenic chemotherapy.^10,11^ It remains controversial whether an NK-1 RA–containing prophylactic regimen should be used in moderately emetogenic chemotherapy of various types of cancers. In a multinational phase 3 study, the aprepitant regimen showed superior efficacy compared with the nonaprepitant regimen in preventing CINV with no vomiting during the overall phase (83.2% vs 71.3%; *P* < .05).^12^ In a Korean phase 4 clinical trial, the proportion of patients with no vomiting while receiving the aprepitant regimen was numerically, but not statistically significantly, higher than with the control regimen (77.2% vs 72.0%; *P* = .19).^13^ In addition, for prophylaxis of oxaliplatin-induced emesis, combining an NK-1 RA with dexamethasone and a 5-HT3 RA is not recommended by MASCC/EMSO guidelines, but it is recommended for select patients with risk factors by the National Comprehensive Cancer Network guideline.^10,11^ This clinical trial was conducted to evaluate the efficacy and safety of combining aprepitant with dexamethasone and palonosetron in patients with gastrointestinal cancer at higher risk of emesis who were treated with FOLFOX or FOLFIRI.

## Methods

### Study Design

This randomized, double-blind, placebo-controlled phase 3 trial was performed at 4 independent centers in China. All included patients provided written informed consent before registration. The study was approved by the independent ethics committee or institutional review board at each participating center. This study followed the Consolidated Standards of Reporting Trials (CONSORT) reporting guideline. The trial protocol is available in Supplement 1. The efficacy of antiemetic therapy was monitored from the start of chemotherapy agent infusion (0 hours on day 1) to the morning of day 6 (approximately 120 hours). Patients were instructed to complete the patient diary to report episodes of emesis, the use of rescue therapy, and assessment of daily nausea and vomiting by a 100-mm visual analog scale. Any episode of vomiting or retching and use of rescue therapy was to be recorded. On day 6, patients needed to immediately complete the Functional Life Index–Emesis (FLIE) questionnaire concerning days 1 to 5. The FLIE is a measurement tool for evaluating the prevalence of CINV and its association with quality of life. The tool consists of 18 questions, with each scaled from 1 to 7. The first 9 questions are related to the grade and effect of nausea, and the last 9 are related to emesis.^14^ The completed diary was returned to the study assistant at the patient’s next chemotherapy cycle.

Many risk factors are associated with CINV. In this study, we focused on younger age, female sex, and little or no history of alcohol use. Those factors are routinely documented when data on the patient’s history are collected. Women aged 18 to 50 years with no history of long-term or excessive alcohol intake (alcohol intake of <5 times per week and 100 g per day), Eastern Cooperative Oncology Group (ECOG) performance status score of 0 to 2, and a histologically confirmed gastrointestinal carcinoma that was naive to aprepitant and FOLFOX or FOLFIRI regimens were considered eligible. The major exclusion criteria were inability to read, comprehend, and finish questionnaires, including visual analog scale questions; no history of morning sickness during pregnancy; presence of gastrointestinal tract obstruction or electrolyte imbalance; history of central nervous system disease; concomitant therapy with psychotropic medications, such as olanzapine; and hypersensitivity history or contraindication to use of aprepitant, 5-HT3 RAs, or dexamethasone.

Patients were first stratified according to the chemotherapy regimen (FOLFOX or FOLFIRI). Then, they were randomized (1:1) to receive the aprepitant or placebo regimen in accordance with the unique, computer-generated, blinded allocation schedule, which was developed using SAS, version 9.13 (SAS Institute Inc) and maintained with confidentiality. The investigators, study coordinators, and patients remained blinded to the study assignments. In addition, the matched placebo with the same configuration as the aprepitant capsule was used to maintain double-blinding.

### Regimens and Assessment

Aprepitant, 125 mg, or placebo, 125 mg, was orally administered 60 minutes before initiation of chemotherapy on day 1, and aprepitant, 80 mg, or placebo, 80 mg, was administered orally each morning of days 2 and 3. Both groups received palonosetron, 0.25 mg, intravenously and dexamethasone (6 mg in the aprepitant regimen and 12 mg in the placebo regimen) orally 30 minutes before chemotherapy initiation on day 1 (eTable 1 in Supplement 2). If needed, rescue therapy for nausea or emesis was given according to clinical guidelines.

Patients received FOLFOX or FOLFIRI chemotherapy. The FOLFOX regimen consisted of oxaliplatin, 85 mg/m^2^, given as a 2-hour intravenous infusion and leucovorin, 400 mg/m^2^ (or levoleucovorin, 200 mg/m^2^), administered as a 2-hour intravenous infusion, followed by an intravenous bolus dose of fluorouracil, 400 mg/m^2^, and a 46-hour intravenous infusion of fluorouracil, 2400 mg/m^2^. The FOLFIRI regimen consisted of irinotecan, 180 mg/m^2^, administered as a 90-minute intravenous infusion with the same leucovorin and fluorouracil regimens as used with FOLFOX chemotherapy.

The primary end point was the complete response (CR) rate, defined as the proportion of patients without emesis episodes or rescue medication use during the overall phase (0-120 hours). The secondary end points were (1) CR rate in the acute and delayed phases; (2) the proportion of no vomiting (no vomiting or retching episodes) in the acute, delayed, and overall phases; (3) effects of CINV on daily life (considered as no substantial harmful effects on daily life when total points on the FLIE questionnaire were ≥108); and (4) duration to the first episode of vomiting. Exploratory efficacy end points included no substantial nausea (nausea visual analog scale score <25 mm), no nausea (nausea visual analog scale score <5 mm), complete control (no emesis, no rescue therapy, and nausea visual analog scale score <25 mm), total control (no emesis, no rescue therapy, and nausea visual analog scale score <5 mm), and effects of nausea or emesis on daily life (considered as no substantial harmful effect on daily life when the domain score of nausea or emesis in the FLIE questionnaire was ≥54). All criteria were applied to evaluate the acute (0-24 hours), delayed (24-120 hours), and overall (0-120 hours) phases. Adverse events were graded according to the National Cancer Institute Common Toxicity Criteria for Adverse Events, version 4.0. Unless meeting the criteria of the serious adverse events, nausea and vomiting were not reported as adverse events in this study.

### Statistical Analysis

Statistical analysis was conducted on October 30, 2020. In a phase 2 trial comparing the aprepitant group with the placebo group in women younger than 70 years with no alcohol use receiving moderately emetogenic chemotherapy, the CR rates in the overall phase were 62% with aprepitant and 52% with placebo.^15^ Thus, we assumed a CR rate of 50% for the placebo regimen and 70% for the aprepitant regimen. After calculation, 248 participants (124 participants per group) were required under a power of 90% and a 2-sided type I error of 5%.

The modified intent-to-treat population (patients who received chemotherapy and a study treatment and were analyzed according to the allocated group) was used to analyze patient baseline characteristics and efficacy analyses. The safety population (patients who received chemotherapy and a study treatment but were analyzed according to the treatment they received) was used to analyze safety and tolerability.

The differences in baseline characteristics between the 2 groups were analyzed by *t* test for measurement data and by the Pearson χ^2^ test or Fisher exact probability method for classification data. The χ^2^ test or Fisher exact test was also used to analyze the differences between all kinds of efficacy end points. Logistic regression analysis was used for subgroup analysis of predictive factors for CR in the overall phase. Clinical factors with *P* values <.20 on univariable analysis and chemotherapy regimen factors were included in multivariable analysis. A 2-sided *P* value <.05 was regarded as statistically significant. All statistical analyses were performed with SPSS, version 24.0 (IBM Corp).

## Results

### Baseline Characteristics

**Figure 1.**
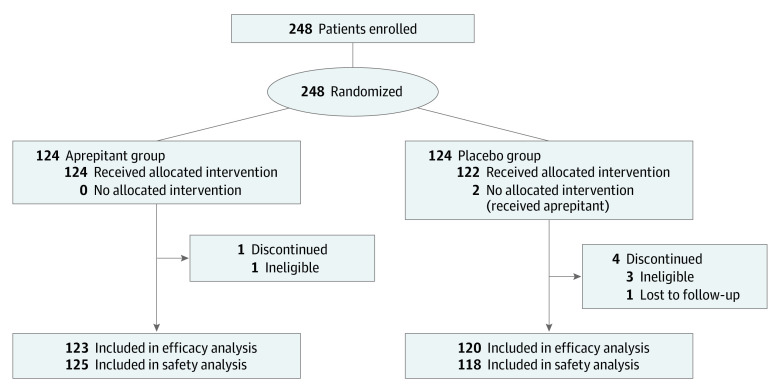
Consolidated Standards of Reporting Trials Diagram

A total of 248 women younger than 50 years from 4 independent centers in China were enrolled between August 4, 2015, and March 31, 2020, and were randomly assigned to an aprepitant or placebo regimen in the 1:1 ratio (Figure 1). After randomization, 1 patient in the aprepitant group and 3 patients in the placebo group failed to meet eligibility criteria (4 patients with cancers other than gastrointestinal cancer were mistakenly enrolled); 1 patient in the placebo group was lost to follow-up. These individuals were consequently excluded from the efficacy analyses. Two patients in the placebo group purchased and used aprepitant not in accordance with the regimen. A total of 125 patients received an aprepitant regimen and 118 patients received a placebo regimen; this was considered the safety population and used to analyze safety and tolerability.

The baseline characteristics were similar in the 2 treatment groups (Table 1). There were no significant differences in age, body surface area, body mass index, history of previous chemotherapy, ECOG performance status score, the tumor’s primary site, and TNM stage between the 2 groups. The mean (SD) age of the total population was 40.1 (7.3) years (range, 20-50 years). One hundred eighty-one patients (74.5%) had colorectal cancer and 179 patients (73.7%) had stage IV gastrointestinal cancer. One hundred ninety-three patients (79.4%) received FOLFOX chemotherapy and 50 patients (20.6%) received FOLFIRI chemotherapy.

**Table 1.** Baseline Characteristics

Characteristic	Patients, No. (%)	
	Aprepitant group (n = 123)	Placebo group (n = 120)
Age, mean (SD) [range], y	40.01 (7.42) [20-50]	40.17 (7.27) [21-50]
Asian race	123 (100)	120 (100)
Previous chemotherapy		
Yes	87 (70.7)	79 (65.8)
No	36 (29.3)	41 (34.2)
BSA, mean (SD), m^2^	1.53 (0.13)	1.53 (0.12)
BMI, mean (SD)	20.82 (3.84)	20.67 (2.89)
ECOG performance status score		
0	33 (26.8)	28 (23.3)
1	83 (67.5)	86 (71.7)
2	7 (5.7)	6 (5.0)
Primary site of tumor		
Stomach	23 (18.7)	30 (25.0)
Small intestine	3 (2.4)	6 (5.0)
Colorectum	97 (78.9)	84 (70.0)
TNM stage		
II	5 (4.1)	0
III	32 (26.0)	27 (22.5)
IV	86 (69.9)	93 (77.5)
Chemotherapy regimen		
FOLFOX	98 (79.7)	95 (79.2)
FOLFIRI	25 (20.3)	25 (20.8)

Abbreviations: BMI, body mass index (calculated as weight in kilograms divided by height in meters squared); BSA, body surface area; ECOG, Eastern Cooperative Oncology Group; FOLFIRI, fluorouracil, leucovorin, and irinotecan; FOLFOX, fluorouracil, leucovorin, and oxaliplatin.

### Efficacy

**Figure 2.**
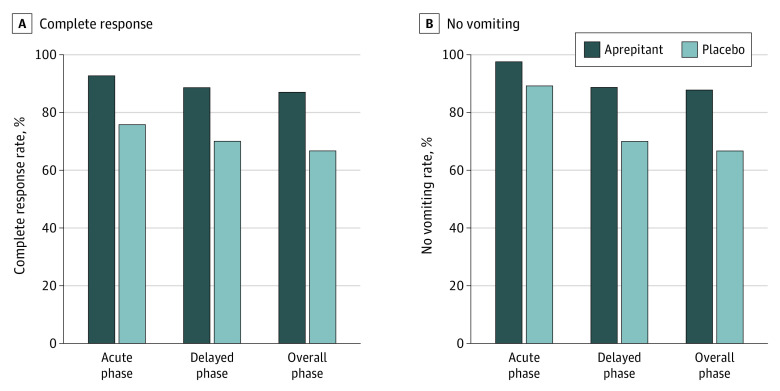
Proportion of Patients Achieving Complete Response or No Vomiting Overall and by Study Phase A, Proportion of patients achieving complete response in the aprepitant and placebo groups during the acute, delayed, and overall phases. B, Proportion of patients without vomiting in the aprepitant and placebo groups during the acute, delayed, and overall phases.

Assessing the primary efficacy end point in the modified intention-to-treat population, 107 of 123 patients (87.0%) in the aprepitant group and 80 of 120 patients (66.7%) in the placebo group achieved the CR in the overall phase (*P* < .001). The CR rates were 114 (92.7%) vs 91 (75.8%) in the acute phase (*P* < .001) and 109 (88.6%) vs 84 (70.0%) in the delayed phase (*P* < .001) (Figure 2A). In addition, in the 193 patients who received FOLFOX chemotherapy, the CR rate in the overall phase was significantly higher in the aprepitant vs placebo group (88 [89.8%] vs 63 [66.3%]; *P* < .001); in the 50 patients who received FOLFIRI chemotherapy, the CR rate in the overall phase was numerically, but not significantly, higher (19 [76.0%] vs 17 [68.0%]; *P* = .53) (eFigure in Supplement 2). The no-vomiting rate was significantly higher in the aprepitant group than in the placebo group (acute phase: 120 [97.6%] vs 107 [89.2%]; *P* = .008; delayed phase: 109 [88.6%] vs 84 [70.0%]; *P* < .001; and overall phase: 108 [87.8%] vs 80 [66.7%]; *P* < .001) (Figure 2B).

There were significant differences in other efficacy end points (Table 2). The proportion of patients with no nausea or significant nausea in the overall phase was higher in the aprepitant vs placebo group (no nausea: 81 [65.9%] vs 59 [49.2%]; *P* = .008 [16.7–percentage point difference]; significant nausea: 121 [98.4%] vs 111 [92.5%]; *P* = .03 [5.9–percentage point difference]). The FLIE questionnaire showed that more patients in the aprepitant group reported a less negative influence of CINV on daily life within the 5-day measurement period. Quality of life in the aprepitant vs placebo group was significantly better according to the FLIE score (107 [87.0%] vs 80 [66.7%]; *P* < .001), nausea domain score (101 [82.1%] vs 76 [63.3%] [18.8–percentage point difference]; *P* = .001), and vomiting domain score (112 [91.1%] vs 82 [68.3%] [22.8–percentage point difference]; *P* < .001).

**Table 2.** Secondary and Exploratory Efficacy End Points in 2 Groups

End point	Patients, No. (%)		*P* value
	Aprepitant group (n = 123)	Placebo group (n = 120)	
No significant nausea, overall phase	121 (98.4)	111 (92.5)	.03
No nausea, overall phase	81 (65.9)	59 (49.2)	.01
Complete control, overall phase	107 (87.0)	80 (66.7)	<.001
Total control, overall phase	77 (62.6)	55 (45.8)	.01
FLIE total scores, ≥108	107 (87.0)	80 (66.7)	<.001
Nausea domain, ≥54	101 (82.1)	76 (63.3)	.001
Emesis domain, ≥54	112 (91.1)	82 (68.3)	<.001

Abbreviation: FLIE, Functional Life Index–Emesis.

**Figure 3.**
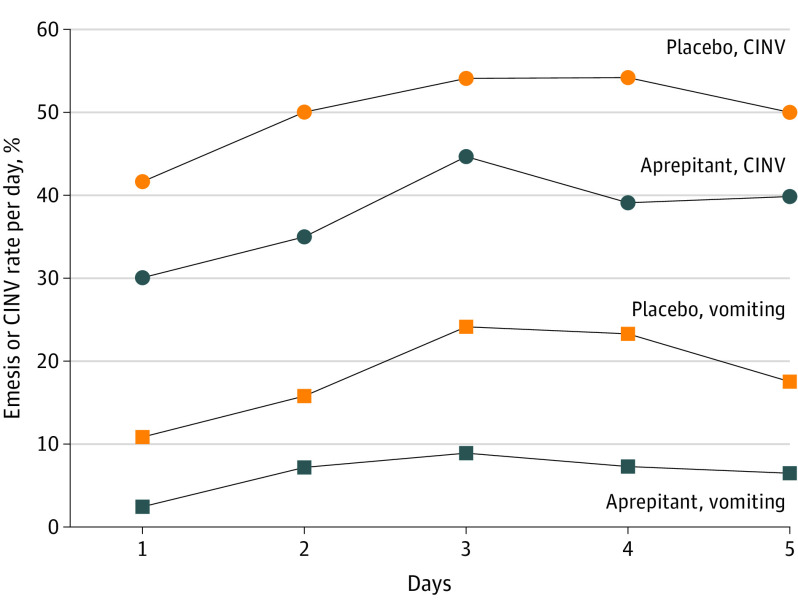
The Proportion of Patients With Emesis or Chemotherapy-Associated Nausea and Vomiting (CINV) per Day

Figure 3 shows the proportion of patients with CINV or emesis per day. In the overall phase, the rate of emesis or CINV remained lower in the aprepitant group per day. The CINV proportion was highest on the third day (aprepitant: 44.7%; placebo: 54.2%). On the fifth day, 39.8% of patients receiving aprepitant vs 50.0% of patients receiving still had CINV, including 6.5% of those receiving aprepitant vs 17.5% of patients with emesis.

### Safety and Tolerability

The incidence of at least 1 adverse event was generally similar in patients treated with the aprepitant (80.0%) and placebo (81.3%) regimens (*P* = .79) (eTable 2 in Supplement 2). Myelosuppression, transaminase level elevation, anorexia, peripheral neurotoxicity, and fatigue were the most frequently reported adverse events in both the aprepitant and placebo groups. A grade 3 or 4 adverse event was reported in 25 of 125 patients (20.0%) in the aprepitant group and 15 of 118 patients (12.7%) in the placebo group (*P* = .13). In addition, 2 patients in each group developed grade 4 neutropenia and 1 patient in the placebo group had grade 4 leukopenia. Except for the hematologic and biochemical blood indexes, 3 patients (2.4%) in the aprepitant group and 1 patient (0.8%) in the placebo group had grade 3 diarrhea. None of the grade 3 or 4 adverse events was considered to be associated with aprepitant by the attending clinician. No grade 5 adverse events were reported in either of the groups.

### Risk Factors for CR

The results of univariable and multivariable analyses of the risk factors associated with CR in the overall phase are reported in eTable 3 in Supplement 2. Patients’ baseline characteristics, chemotherapy regimen, and aprepitant use were included in the analysis. In the univariable analysis, only the use of aprepitant (odds ratio, 3.34; 95% CI, 1.75-6.39; *P* < .001) had a *P* value <.10. Thus, clinical factors (age, history of previous chemotherapy, tumor’s primary site, and ECOG performance status score) at *P* < .20 on univariable analysis and chemotherapy regimen factors were included in multivariable analysis. The multivariable analysis indicated that only aprepitant use was a significant risk factor (odds ratio, 3.42; 95% CI, 1.77-6.61; *P* < .001).

## Discussion

The addition of an NK-1RA in the prophylaxis of CINV in patients receiving moderately emetogenic chemotherapy remains controversial.^16,17^ In previous studies, the NK-1 RA showed better antiemetic efficacy in patients treated with specific moderately emetogenic chemotherapy regimens, such as anthracycline with cyclophosphamide, which are now categorized as highly emetogenic chemotherapy.^18^ For the regimen not including anthracycline and cyclophosphamide, clinical studies have shown conflicting results on the efficacy of the combined use of aprepitant for improving nausea and emesis in various moderately emetogenic chemotherapy regimens and tumor types.^12,13,15,19^

The FOLFOX and FOLFIRI regimens have shown survival benefits in treatment of gastrointestinal cancer; however, their use also increased the incidence of CINV compared with fluorouracil alone.^20,21^ Moreover, patients with gastrointestinal cancers seem more susceptible to emesis owing to the abdominal pain and gastrointestinal obstruction caused by the tumor. Nevertheless, the conclusions of 2 large randomized trials showed conflicting outcomes on the role of NK-1 RA in oxaliplatin-based chemotherapy.^22,23^ The first trial reported no statistically significant differences in the rate of CR in the NK-1 RA group (casopitant, ondansetron, and dexamethasone) compared with the placebo group (placebo, ondansetron, and dexamethasone) for the overall (86% vs 85%; *P* = .73), acute (97% vs 96%; *P* = .48), or delayed (86% vs 85%; *P* = .73) phases.^22^ The second trial demonstrated that significantly more patients in the NK-1 RA group (aprepitant plus fosaprepitant and 5-HT3 RA plus dexamethasone) achieved CR compared with the control group (5-HT3 RA plus dexamethasone) overall (85.0% vs 74.3%; *P* = .01) and for the delayed phase (85.0% vs 75.4%; *P* = .02), but not for the acute phase (94.7% vs 92.4%; *P* = .37).^23^ In addition, the first trial did not show the subgroup analysis of risk factors for CINV, but the second trial found that aprepitant was more effective in the prevention of CINV among women.^24^ Based on the available evidence, the National Comprehensive Cancer Network guideline and MASCC/ESMO guideline did not reach full agreement on the role of NK-1 RAs in moderately emetogenic chemotherapy. The MASCC/ESMO guideline does not recommend combining an NK-1 RA for the prophylaxis of oxaliplatin-induced CINV, but the National Comprehensive Cancer Network guideline suggests that oxaliplatin and irinotecan may be highly emetogenic in certain patients and an NK-1 RA should be added to a dexamethasone and 5-HT3 antagonist regimen for these selected patients.

These additional risk factors, such as younger age and female sex, were assessed and an association was shown in many cross-sectional studies and subgroup analyses that revealed numerous inconsistencies. A European prospective observational study clarified the contribution of those risk factors in CINV development in variable chemotherapy regimens and antiemetic regimens.^25^ Thus, the National Comprehensive Cancer Network guideline affirms the use of NK-1 RA in specific patients receiving moderately emetogenic chemotherapy. However, to our knowledge, only a few prospective clinical studies^12,13,15,18^ have been conducted to evaluate the antiemetic efficacy of NK-1 RA, especially for high-risk patients, and, most importantly, it remains unclear how much such patients could benefit from the combined use of NK-1 RA in prophylactic treatment strategies.

Our study focused on patients with higher risk factors for CINV, including younger age, female sex, and little or no history of alcohol use. Those factors are easy to rapidly and accurately identify when documenting the patient’s history. The results indicated that improvement of the control of emesis was achieved with aprepitant vs placebo overall (87.0% vs 66.7%; *P* < .001) and in the acute (92.7% vs 75.8%, *P* = .001) and delayed (88.6% vs 70.0%; *P* = .001) phases. Although palonosetron has a stronger binding affinity for the 5-HT3 receptor and a longer half-life compared with ondansetron and granisetron, the 2-drug combination of palonosetron and dexamethasone was not satisfactory in the prevention of CINV; 66.7% of the patients who received placebo reached CR, but 33.3% of the patients did not. With the addition of aprepitant, the CR rate increased to 87.0%. Aprepitant also had an important role in improving protection against nausea. In the overall phase, the incidence of CINV per day in the aprepitant group remained lower than in the placebo group. Increases were seen in the proportion of no nausea (16.7%) and no substantial nausea (5.9%). In addition, the reduction in the incidence of CINV improved the patients’ quality of life. The proportions of a detrimental effect on quality of life decreased by 18.8% for nausea and 22.8% for emesis according to the FLIE questionnaire scores.

In this study, other risk factors, including history of motion sickness and morning sickness during pregnancy, were not included for the following reasons. First, the diagnoses of motion sickness need to be differentiated from other diseases, such as Meniere disease and benign paroxysmal positional vertigo. Second, most pregnant women experience morning sickness during pregnancy, and the severity of morning sickness may be more associated with the risk of CINV, but there was recall bias in this history. Third, those 2 risk factors are not usually mentioned during routine history interviews. To make it easier to conduct the research and generalize the research conclusion to clinical practice, we selected only age, sex, and alcohol use history.

The triple-drug combination of aprepitant plus palonosetron and dexamethasone was well tolerated in general. There was no significant difference in the total incidence of all grade adverse events between the 2 groups. However, compared with the placebo group, the total incidence of grade 3 or 4 adverse events seemed numerically higher in the aprepitant group. Most grade 3 or 4 adverse events were abnormal results of hematologic tests, which were slightly associated with aprepitant. The possibility of hematologic adverse events highlights the importance of reminding patients to accept routine blood tests regardless of the antiemetic regimen and chemotherapy regimen being used.

### Limitations

This study has limitations. First, the prevention of aprepitant-induced CINV was evaluated solely during the first course of chemotherapy. The role of aprepitant in anticipatory vomiting and during the complete treatment regimen is unknown. Second, in the subgroup analysis, with respect to the chemotherapy regimen, the antiemetic effects of adding aprepitant were just numerically (ie, not significantly) better for patients receiving the FOLFIRI regimen (76.0% vs 68.0%, *P* = .53). Only 50 patients received the FOLFIRI regimen in our study; thus, future studies with larger sample sizes are warranted. Third, the inclusion of only women younger than 50 years with little or no alcohol use limits the generalizability of the trial results.

## Conclusions

This study demonstrates increased antiemetic efficacy of the combination of aprepitant with palonosetron and dexamethasone during FOLFOX or FOLFIRI chemotherapy. The aprepitant regimen was well tolerated by younger women with gastrointestinal cancer who had a history of little or no alcohol consumption.

## Notes

Supplement 1. **Trial Protocol**

Supplement 2. **eFigure.** The Complete Response Rate in Patients Receiving Different Chemotherapy Regimens in the Overall Phase **eTable 1.** Antiemetic Treatment Regimens **eTable 2.** Frequency and Percent of Adverse Events **eTable 3.** Predictive Factors Associated With Complete Response in the Overall Phase

Supplement 3. **Data Sharing Statement**
