# Supplementary material for: Effect of Aprepitant for the Prevention of Chemotherapy-Induced Nausea and Vomiting in Women: A Randomized Clinical Trial
Source: JAMA Netw Open. 2021 Apr 9;4(4):e215250. doi: 10.1001/jamanetworkopen.2021.5250 (PMC8035650; doi:10.1001/jamanetworkopen.2021.5250)
Supplement: Supplement 1. — Trial Protocol [file jamanetwopen-e215250-s001.pdf]

Clinical Trial Protocol： Aprepitant Triple Therapy for the Prevention of CINV in Nondrinking and Young Women Who Received Moderately Emetogenic Chemotherapy (ClinicalTrials.gov Identifier: NCT03674294)

Study Description

Brief Summary:

The purpose of this study is to study whether adding aprepitant to palonosetron and dexamethasone can further prevent the incidence and severity of nausea and vomiting caused by FOLFIRI or FOLFOX chemotherapy regimen among gastrointestinal malignancy patients with high risk factors of chemotherapy-associated adverse events.

Detailed Description:

The purpose of this study is to study whether adding aprepitant to palonosetron and dexamethasone can further prevent the incidence and severity of nausea and vomiting caused by FOLFIRI or FOLFOX chemotherapy regimen after curative effect among gastrointestinal malignancy patients with high risk factors of chemotherapy-associated adverse events. This study will observe and evaluate the incidence and severity of nausea and vomiting as well as the effectiveness of corresponding treatment (with or without aprepitant) during Day 1 to Day 5 from the beginning of chemotherapy.

Study Design

Study Type: Interventional (Clinical Trial)

Estimated Enrollment: 248 participants

Allocation: Randomized

Intervention Model: Parallel Assignment

Masking: Double (Participant, Investigator)

Primary Purpose: Prevention

Official Title: Efficacy of aprepitant for the prevention of chemotherapy-induced nausea and vomiting in nondrinking women younger than 50 years who received moderately emetogenic chemotherapy: A randomized, double-blind, phase III trial

Study Start Date: August 4, 2015

Primary Completion Date: March 31, 2020

Study Completion Date: June 1, 2020

Outcome Measures

Primary Outcome Measures:

Complete response rate during the overall phase

Secondary Outcome Measures:

- CR rate in the acute and delayed phase
- The proportion of no vomiting in the acute phase, delayed phase and overall phase
- Effects of CINV on daily life
- Duration to the first time of vomiting

Exploratory Outcome Measures:

- No significant nausea
- No nausea
- Complete control
- Total control
- Effects of nausea or emesis on daily life

Statistical Methodology

In a phase II trial comparing the aprepitant group with the placebo group in non-drinking women younger than 70 years receiving moderately emetogenic chemotherapy, the CR rates in the overall phase were 62% and 52% respectively15. Thus, we assumed a CR rate of 50% in the placebo regimen and 70% in the aprepitant regimen. After calculation, 248 participants (124 participants per group) were required under a power of 90% and a two-sided type I error of 5%.

The modified intent-to-treat (mITT) population (patients who received chemotherapy and a study treatment and were analyzed according to the allocated group) was used to analyze patient baseline characteristics and efficacy analyses. Safety population (patients who received chemotherapy and a study treatment but were analyzed according to the treatment they actually received) was used to analyze safety and tolerability.

The differences of baseline characteristics between the two groups were analyzed by t-test for measurement data and by person's $\chi 2$ test or Fisher's exact probability method for classification data. The $\chi 2$ test or Fisher's exact test was also used to analyze the differences between all kinds of efficacy endpoints. Logistic regression analysis was used for subgroup analysis of predictive factors for CR in the overall phase. Clinical factors with P values less than 0.20 on univariate analysis and chemotherapy regimen factors were included in multivariate analysis. A two-sided P-value less than 0.05 was regarded as statistically significant. All statistical analyses were performed with the IBM SPSS Statistics 24.0 (IBM, Armonk, NY, USA).

Eligibility Criteria

Inclusion Criteria:

- Diagnosed by pathology as gastrointestinal carcinoma and no previous FOLFOX or FOLFIRI based regimen chemotherapy history
- Female
- Adult patients ($\geq$ 18, $\leq$ 50 years of age)
- No long-term or excessive alcohol intake history:1. Alcohol intake less than 5 times per week; 2. Alcohol intake less than 100g per day
- Performance status ECOG 0-1
- Adequate hematological, hepatic, renal and metabolic function parameters:

Leukocytes: 3,500-10,000/mm3, ANC $\geq$ 1,500/mm3, Platelets $\geq$ 90,000/mm3, Hb > 9g/dl (may be transfused or treated with erythropoietin to maintain or exceed this level), Serum creatinine $\leq$ 1 x upper

limit of normal, Bilirubin ≤ 1.5 x upper limit of normal, Serum AST, ALT, ALP ≤ 2.5 x upper limit of normal in absence of liver metastases, or ≤ 5 x upper limit of normal in presence of liver metastases.

- Negative pregnancy test. If pregnancy test were positive, subject should be included in the trial only when the subsequent pregnancy test is negative
- Ability of reading, comprehending and finishing trial questionnaires and record, including VAS question
- Before subject registration, written informed consent must be given according to local regulations

Exclusion Criteria:

- Pregnant women without morning sickness
- Presence of gastrointestinal tract obstruction or electrolyte imbalance
- Any history of central nervous system disease (e.g. Primary brain tumor, seizure not controlled with standard medical therapy, brain metastases or history of stroke)
- Contraindication of glucocorticoid:1. Infection of virus, bacteria or fungus uncontrolled by antibiotics; 2. Active stomach or duodenum ulcer; 3. Severe hypertension, atherosclerosis, diabetes; 4. Osteoporosis;5. Corneal ulcer; 6. Pregnancy; 7. Reparative phase of trauma, operation or fraction; 8. Hypercortisolism; 9. Severe mental disorder or epilepsy; 10. Inadequate cardiac or renal function
- Mental disability or severe emotional or mental disorder
- Active infection (e.g. pneumonia, hepatitis) or any uncontrolled disease (e.g. diabetic ketoacidosis) that may affect study outcome or expose patients to unnecessary risk
- Usage of any illicit drug, including medical marijuana or alcohol abusing (China drug dependence criteria)
- Treatment of unapproved medicine in the previous 4 weeks
- Concomitant therapy of psychotropic medicine such as olanzapine
- Hypersensitivity history towards Aprepitant, 5-HT3 receptor antagonist or dexamethasone
- Previous treatment of Aprepitant
- Unable to swallow capsules
- Main researchers considered that the patient is unsuited to the trial
- Unable or unwilling to follow research program
