# Supplementary material for: Effect of Aprepitant for the Prevention of Chemotherapy-Induced Nausea and Vomiting in Women: A Randomized Clinical Trial
Source: JAMA Netw Open. 2021 Apr 9;4(4):e215250. doi: 10.1001/jamanetworkopen.2021.5250 (PMC8035650; doi:10.1001/jamanetworkopen.2021.5250)
Supplement: Supplement 2. — eFigure. The Complete Response Rate in Patients Receiving Different Chemotherapy Regimens in the Overall Phase eTable 1. Antiemetic Treatment Regimens eTable 2. Frequency and Percent of Adverse Events eTable 3. Predictive Factors Associated With Complete Response in the Overall Phase [file jamanetwopen-e215250-s002.pdf]

Supplementary Online Content

Wang DS, Hu MT, Wang ZQ, et al. Effect of aprepitant for the prevention of chemotherapy-induced nausea and vomiting in women: a randomized clinical trial. *JAMA Netw Open*. 2021;4(4):e215250. doi:10.1001/jamanetworkopen.2021.5250

eFigure. The Complete Response Rate in Patients Receiving Different Chemotherapy Regimens in the Overall Phase

eTable 1. Antiemetic Treatment Regimens

eTable 2. Frequency and Percent of Adverse Events

eTable 3. Predictive Factors Associated With Complete Response in the Overall Phase

This supplementary material has been provided by the authors to give readers additional information about their work.

eFigure. The complete response rate in patients receiving different chemotherapy regimens in the overall phase.

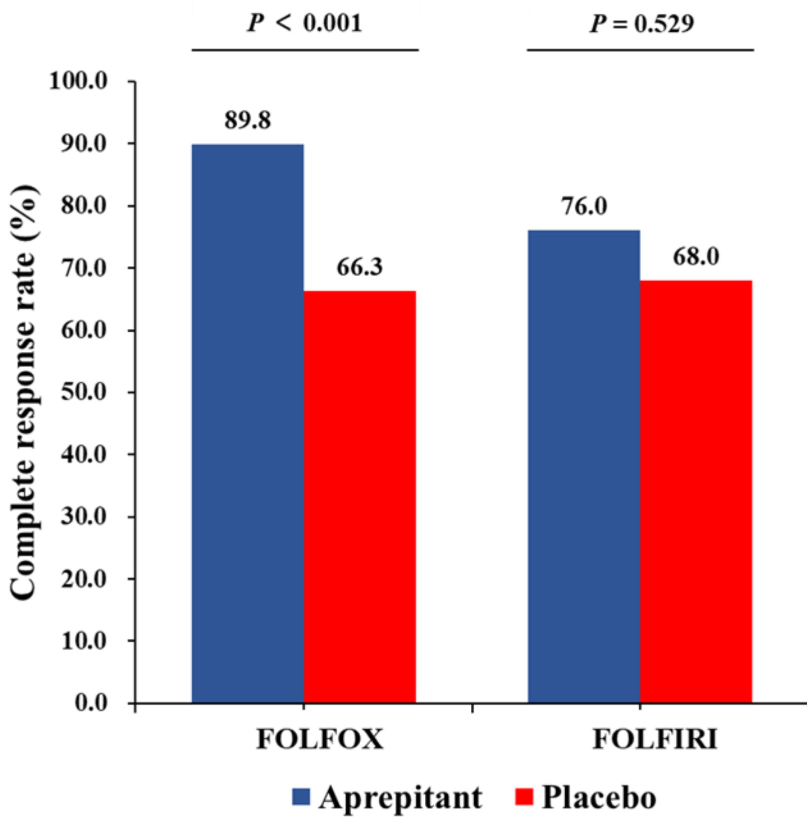

© 2021 Wang DS et al. *JAMA Network Open.*

eTable 1. Antiemetic treatment regimens.

Regimen	Drugs	D1	D2	D3
Aprepitant	Aprepitant	125mg p.o., 60 min before chemotherapy	80mg p.o.	80mg p.o.
	Palonosetron	0.25mg i.v., 30 min before chemotherapy	None	None
	Dexamethasone	6mg p.o., 30 min before chemotherapy	None	None
Placebo	Placebo	125mg p.o., 60 min before chemotherapy	80mg p.o.	80mg p.o.
	Palonosetron	0.25mg i.v., 30 min before chemotherapy	None	None
	Dexamethasone	12mg p.o., 30 min before chemotherapy	None	None

Abbreviations: p.o., oral administration ; i.v., intravenous injection.

© 2021 Wang DS et al. *JAMA Network Open.*

eTable 2. Frequency and percent of adverse events.

	No. (%)						
	Aprepitant group (n = 125)				Placebo group (n = 118)		
	All	Grade 3	Grade 4		All	Grade 3	Grade 4
**≥1 AE**							
Yes	100 (80.0)	25 (20.0)	2 (1.6)		96 (81.3)	13 (11.0)	3 (2.5)
**Hematological**							
Leukopenia	27 (21.6)	4 (3.2)	0 (0)		29 (24.6)	2 (1.6)	1 (0.8)
Neutropenia	44 (35.2)	13 (10.4)	2 (1.6)		41 (34.7)	7 (5.9)	2 (1.6)
Anemia	48 (38.4)	6 (4.8)	0 (0)		59 (50.0)	3 (2.5)	0 (0)
Thrombocytopenia	2 (1.6)	0 (0)	0 (0)		3 (2.5)	0 (0)	0 (0)
**Metabolic**							
Alkaline phosphatase elevation	10 (8.0)	0 (0)	0 (0)		9 (7.6)	0 (0)	0 (0)
Alanine aminotransferase elevation	11 (8.8)	0 (0)	0 (0)		7 (5.9)	0 (0)	0 (0)
Aspartate aminotransferase elevation	13 (10.4)	1 (0.8)	0 (0)		11 (9.3)	1 (0.8)	0 (0)
Blood bilirubin elevation	1 (0.8)	0 (0)	0 (0)		4 (3.4)	0 (0)	0 (0)
Creatinine elevation	1 (0.8)	0 (0)	0 (0)		0 (0)	0 (0)	0 (0)
**General**							
Fever	0 (0)	0 (0)	0 (0)		2 (1.7)	0 (0)	0 (0)
Fatigue	10 (8.0)	0 (0)	0 (0)		9 (7.6)	0 (0)	0 (0)
Weight loss	9 (7.2)	0 (0)	0 (0)		8 (6.8)	0 (0)	0 (0)
**Gastroenterological**							
Anorexia	27 (21.6)	0 (0)	0 (0)		19 (16.1)	0 (0)	0 (0)
Diarrhea	8 (6.4)	3 (2.4)	0 (0)		11 (9.3)	1 (0.8)	0 (0)
Constipation	9 (7.2)	0 (0)	0 (0)		6 (5.1)	0 (0)	0 (0)
Abdominal pain	1 (0.8)	0 (0)	0 (0)		2 (1.7)	0 (0)	0 (0)
Anal pain	1 (0.8)	0 (0)	0 (0)		0 (0)	0 (0)	0 (0)

© 2021 Wang DS et al. *JAMA Network Open.*

Continued

	Aprepitant group (n = 125, %)				Placebo group (n = 118, %)		
	**All**	**Grade 3**	**Grade 4**		**All**	**Grade 3**	**Grade 4**
Mucositis oral	1 (0.8)	0 (0)	0 (0)		4 (3.4)	0 (0)	0 (0)
Dry mouth	1 (0.8)	0 (0)	0 (0)		0 (0)	0 (0)	0 (0)
**Dermatologic**							
Rash	3 (2.4)	0 (0)	0 (0)		5 (4.2)	0 (0)	0 (0)
Alopecia	1 (0.8)	0 (0)	0 (0)		3 (2.5)	0 (0)	0 (0)
Skin hyperpigmentation	1 (0.8)	0 (0)	0 (0)		0 (0)	0 (0)	0 (0)
**Immunological**							
Anaphylaxis	1 (0.8)	0 (0)	0 (0)		0 (0)	0 (0)	0 (0)
**Neurologic**							
Peripheral neurotoxicity	12 (9.6)	0 (0)	0 (0)		10 (8.5)	0 (0)	0 (0)
Dizziness	0 (0)	0 (0)	0 (0)		2 (1.7)	0 (0)	0 (0)

© 2021 Wang DS et al. *JAMA Network Open.*

eTable 3. Predictive factors associated with complete response in the overall phase.

	Number of patients	Complete response (n, %)		Univariant analysis			Multivariate analysis	
				OR	*P*-value		OR	*P*-value
**Age (years)**								
＞45	78	64 (82.1)		1			1	
≤45	165	123 (74.5)		0.64 (0.33-1.26)	0.20		0.61 (0.30-1.24)	0.18
**Previous chemotherapy**								
Yes	77	57 (74.0)		1				
No	166	130 (78.3)		1.27 (0.68-2.38)	0.46			
**BSA**								
＞1.55	94	77 (81.9)		1			1	
≤1.55	149	110 (73.8)		0.62 (0.33-1.18)	0.15		0.66 (0.34-1.28)	0.22
**BMI**								
＞19.2	162	121 (74.7)		1				
≤19.2	81	66 (81.5)		1.49 (0.77-2.89)	0.24			
**ECOG PS score**					0.36			0.42
0	61	49 (80.3)		1				
1	169	130 (76.9)		0.82 (0.40-1.69)	0.58		0.88 (0.41-1.88)	0.75
2	13	8 (61.5)		0.39 (0.11-1.41)	0.15		0.41 (0.11-1.58)	0.20
**Primary site of tumor**					0.23			
Stomach	53	39 (73.6)		1				
Small intestine	9	5 (55.6)		0.45 (0.11-1.91)	0.28			
Colorectum	181	143 (79.0)		1.35 (0.67-2.74)	0.41			
**TNM stage**					0.96			
II	5	4 (80.0)		1				
III	59	46 (78.0)		0.89 (0.09-8.62)	0.92			
IV	179	137 (76.5)		0.82 (0.09-7.50)	0.86			

© 2021 Wang DS et al. *JAMA Network Open.*

Continued

	Number of patients	Complete response (n, %)		Univariant analysis			Multivariate analysis	
				OR	*P*-value		OR	*P*-value
Chemotherapy regimen								
FOLFOX	193	151 (78.2)		1			1	
FOLFIRI	50	36 (72.0)		0.72 (0.35-1.45)	0.35		0.66 (0.31-1.42)	0.29
Aprepitant								
No	120	80 (66.7)		1			1	
Yes	123	107 (87.0)		3.34 (1.75-6.39)	＜0.001		3.42 (1.77-6.61)	＜0.001

Abbreviations: SD, standard deviation; BSA, body surface area; BMI, body mass index; ECOG PS score, Eastern Cooperative Oncology Group (ECOG) performance status (PS) score.

© 2021 Wang DS et al. *JAMA Network Open.*
