# Supplementary material for: Effect of Aprepitant for the Prevention of Chemotherapy-Induced Nausea and Vomiting in Women: A Randomized Clinical Trial
Source: JAMA Netw Open. 2021 Apr 9;4(4):e215250. doi: 10.1001/jamanetworkopen.2021.5250 (PMC8035650; doi:10.1001/jamanetworkopen.2021.5250)
Supplement: Supplement 3. — Data Sharing Statement [file jamanetwopen-e215250-s003.pdf]

Data Sharing Statement

Wang. Effect of Aprepitant for the Prevention of Chemotherapy-Induced Nausea and Vomiting in Women. *JAMA Netw Open*. Published April 09, 2021. doi:10.1001/jamanetworkopen.2021.5250

Data
Data available: No
